# The influence of sensorimotor experience on beauty evaluation of preschool children

**DOI:** 10.3389/fnhum.2023.1138420

**Published:** 2023-07-10

**Authors:** Martina Ardizzi, Francesca Ferroni, Aurora Manini, Claudia Giudici, Elena Maccaferri, Stefano Uccelli, Maria Alessandra Umiltà

**Affiliations:** ^1^Unit of Neuroscience, Department of Medicine and Surgery, University of Parma, Parma, Italy; ^2^Department of Biomedical, Metabolic and Neural Sciences, University of Modena and Reggio Emilia, Modena, Italy; ^3^Reggio Children s.r.l., Reggio Emilia, Italy; ^4^Scuole e Nidi d’Infanzia, Reggio Emilia, Italy; ^5^Department of Psychology, University of Milano-Bicocca, Milan, Italy; ^6^Department of Food and Drugs, University of Parma, Parma, Italy; ^7^The Italian Academy for Advanced Studies, Columbia University, New York, NY, United States

**Keywords:** aesthetics, development, embodiment, simulation, mirror mechanisms

## Abstract

Nowadays there is a broad consensus on the role of multimodality in the construction of an embodied aesthetic experience in adults, whereas little is known about the relationship between sensorimotor and aesthetic experience during development. To fill this gap, the present study investigated whether sensorimotor experience with sculpting natural materials (i.e., clay or sand) influences beauty judgments offered to abstract artifacts made by the same materials. Five years old children (n.47) were asked to rate tactile (How smooth is it?), visual (How dark is it?) and beauty (How much do you like it?) proprieties of two artifacts using a visual-analog measurement-tool *ad hoc* developed to fit children’s cognitive skills. Participants rated the artifacts before and after a free-hands manipulation with only one of the two sculpting materials, either sand or clay. Results showed that the greater the sensorimotor interaction experienced with the artifacts, the higher the increment of beauty rating offered to the artifacts made by the same material previously manipulated. No modulations were found for tactile and visual ratings. These results demonstrate that, even in pre-school children, aesthetic experience is specifically linked to its sensorimotor component, supporting, from a developmental perspective, the definition of aesthetic experience as intrinsically rooted on beholders’ bodily experience.

## 1. Introduction

Aesthetic experience represents a unique condition in human perception as, in this case, object perception is inherently linked to the appreciation of its properties rather to the finalistic propensity to act on it. From a neuroscientific perspective, aesthetic experience can be conceived as the state allowing a beholder to “perceive-feel-sense” an object (Di Dio and Gallese, 2009), and involves a rich interplay between brain networks linked to perception, reward, and cognition (Chatterjee and Vartanian, 2014). It is now well established that aesthetic experience, although often directed toward judgment of appraisal, is not completely divorced from sensorimotor component. Indeed, a critical contribution to aesthetic evaluation derives from the activation of embodied mechanisms in response to the viewed stimulus encompassing the simulation of actions, emotions, and corporeal sensations (Freedberg and Gallese, 2007; Siri et al., 2018). Large evidence, collected among adults, has demonstrated that the simulation of the artistic gestures composing an abstract work of art (Leder et al., 2012; Ticini et al., 2014) or the mimicry of facial expressions portrayed in figurative artworks (Ardizzi et al., 2020a,^2021^) increased the aesthetic judgment of observers. In a recent TMS study, by using stimuli depicting static or dynamic representational paintings of human figures or landscapes, it has been shown a link—mediated by dynamism impression—between the amplitude of observers’ motor evoked potentials and their liking judgments (Fiori et al., 2020). This automatic sensorimotor simulation constitutes a basic and universal component of the triadic description of aesthetic experience allowing the processing of elemental features of aesthetic objects as well as their recognition and engagement through embodied mechanisms. Although these processes have been extensively demonstrated in adult populations, no studies to date have investigated whether sensorimotor simulation can participate to the formation of an aesthetic experience in children. Over the past decades there has been an uptick in developmental research demonstrating the presence of spontaneous sensorimotor simulation responses early in life. The youngest sample in which sensorimotor simulation was observed through mu rhythm desynchronization in response to action observation were 4-month-olds (Virji-Babul et al., 2012). Differently, a much earlier debut of sensorimotor engagement has been estimated by using behavioral measures (Meltzoff and Moore, 1989). In general, studies focusing on pre-school populations confirm the presence of spontaneous sensorimotor simulation, producing consistent and convergent results, and linking such responses to action understanding and communication (Salo et al., 2019). Nevertheless, no studies have explored the link between sensorimotor simulation and the formation of an aesthetic experience in pre-school children. Indirect evidence supporting the thesis of a sensorimotor involvement in children’s aesthetic experience comes from studies demonstrating that at 4 years of age, children’s beauty preference has been tied to their personal experience (Parsons et al., 1978; Savva, 2003; Savva and Trimis, 2005). Furthermore, from 3 to 5 years of age, sensitive “micro-developmental” phases within body aesthetic preference have been described (Di Dio et al., 2018). To date, a study directly testing whether children’s aesthetic experience can be influenced by sensorimotor formats is still missing. To fill this gap, in the present study, we collected children’s beauty and sensory ratings to two abstract artifacts made by two different sculpting natural materials (sand and clay) before and after a sensorimotor interaction with only one of two materials. Children were asked to freely explore one of the two materials with their hands. If sensorimotor interaction plays a role in beauty judgment formation, we expect a correlation between the amount of sensorimotor interaction and the modulation of the beauty judgment.

## 2. Materials and methods

The study was conducted in accordance with the Declaration of Helsinki (2013) and was approved by the Institutional Review Board of the University of Parma (Prot. 0009293). Children’s parents or legal representatives provided informed consent to participate in the study.

The study consisted of two phases involving two groups of children enrolled in two consecutive school years. All the children involved came from three different kindergartens in the municipality of Reggio-Emilia and were recruited thanks to the collaboration with Reggio Children Foundation and “Istituzione Scuole e Nidi dell’Infanzia.” All phases of the study were designed in close collaboration with pedagogues, educators, and atellierists. Interaction between experimenters and children were done under the supervision of educators. The whole study was done inside the schools, so a familiar setting for the children that allowed their free and active collaboration.

The first phase of the study (see sections “3.1. Measurement tool development” and “3.2. Measurement tool validation”) was devoted to the design, realization and testing of a visual-analog measurement tool enabling pre-school children to make judgments on a continuous scale. The second phase of the study (see section “3.3. Experimental session”) implemented this tool in an experimental protocol aimed at testing whether sensorimotor interaction can modulate beauty judgment of pre-school populations.

### 2.1. Measurement tool development

To overcome limitations faced by previous studies (Danko-McGhee and Slutsky, 2011; Rodway et al., 2016; Schabmann et al., 2016), we developed a measurement tool allowing preschool children to provide quantitative judgments in line with their cognitive skills.

#### 2.1.1. Participants

During the school year 2019/2020, 60 kindergarten students (mean age = 5.4 years, ± 3 months; *M* = 27) were recruited to develop the measurement tool to be used in the next experimental session.

#### 2.1.2. Procedure

The educational plan for the first year of the three classes involved a pedagogical work focusing on the concept of measurement to get them used to the concept of measuring the much and the little. Students were introduced to the concept of measurement and gained experience measuring concrete objects with various instruments. Once they were familiarized with this concept, students designed a measuring instrument with the help of pedagogues, educators and atellierists. The classes worked independently during the school year, thus developing three different measurement tools. At the end of the year, the educators with the atellierists synthesized these three solutions into a single version. This final version was then presented to the classes who used it to measure concrete and abstract experiences lived in scholastic context and recreational situations.

#### 2.1.3. Measurement tool description

**FIGURE 1:**
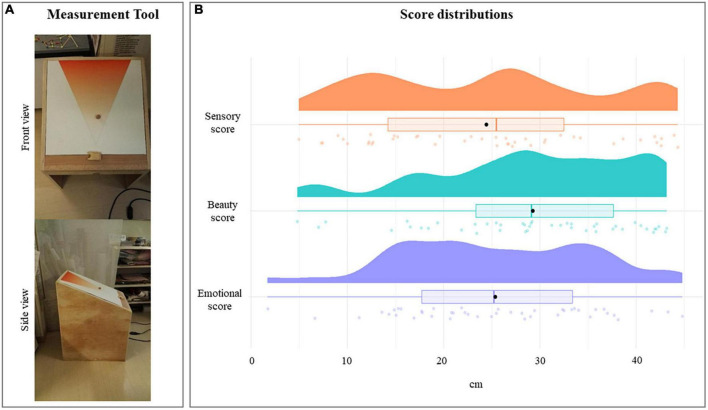
Panel **(A)** front and side views of the *ad hoc* developed measurement tool; panel **(B)** score distributions obtained during measurement tool validation. Black dots indicate the mean values, bold vertical colored lines mark the median values, rectangles identify the interquartile ranges, and the colored areas show scores densities.

The final version of the measurement tool consisted of a white rectangular cardboard (45 cm × 50 cm) resting on a wooden support about 100 cm high on which an inverted isosceles triangle measuring 35 cm × 45 cm was drawn (Figure 1A and Supplementary Video). Throughout its area, the triangle had a lighter color gradient near the vertex (minimum ratings) and a darker one at the base (maximum ratings). The triangle therefore constituted a continuous quantitative scale through which children could provide scores in a visuo-analogic way. The ratings were provided though a wooden circular magnet that could be placed by the children in any area within the triangle. The final version of the measurement tool allowed children to make quantitative judgments in their continuous equivalent, fitting preschool children cognitive development. In fact, literature has shown that preschool children preferably express quantitative estimates through visual-spatial scales, using visual-analogic tools (Sella et al., 2015; Viarouge et al., 2019).

### 2.2. Measurement tool validation

To ensure the validity of the measurement tool created, during the school year 2020/2021 an independent group of children, not involved in measurement tool development, took part in the tool validation.

#### 2.2.1. Participants

During the school year 2020/2021, 44 kindergarten students (mean age = 5.5 years, ± 3 months; *M* = 25) were recruited to test the measurement tool. This group of participants participated also in the experimental session (see below).

#### 2.2.2. Procedure and validation results

After 3 months of familiarization during which the children, accompanied by educators, used the measurement tool to evaluate sensory and emotional everyday experiences, a formal validation of the tool efficacy was performed. Children were asked to use the measurement tool to rate six objects (a puppet, a doll, a photograph of an animal, a song, a candle, and a box of scented tea). Each object was rated according to its sensory (e.g., How smooth is this doll?), beauty (e.g., How much do you like this doll?), and emotional (e.g., How sad is this doll?) proprieties. Figure 1B shows the mean rating and distribution obtained at the three scores. The mean sensory score was 24.43 cm (± 11.67 cm), the mean beauty score was 29.26 cm (± 10.23 cm), whereas the mean emotional score was 25.37 cm (± 10 cm). Score distributions (Figure 1B) revealed that children acquired a good competency in the use of the measurement tool distributing the scores equally among the different scores (sensory vs. beauty two-samples K-S test: *p* = 0.075; sensory vs. emotion two-samples K-S test: *p* = 0.46; emotion vs. beauty two-samples K-S test: *p* = 0.20).

### 2.3. Experimental session

#### 2.3.1. Participants

During the school year 2020/2021, 47 kindergarten students (mean age = 5.5 years, ± 3 months; *M* = 27) were involved in the study. Power was calculated *a posteriori* by means of GLIMMPSE33^1^ using the Hotelling–Lawley Trace which is recommend due to its equivalence to mixed model test. The design included one categorical and two continuous predictors and we checked for main effects and interactions. The actual mean, SD and SD ratio (without scale factor) of the dependent measure was included, together with its real correlation matrix. The significance level was set α = 0.05 resulting in an actual power of 0.87 with our sample size (n.47). Children had normal or corrected-to-normal visual acuity and had no declared developmental disorders.

#### 2.3.2. Procedure

**FIGURE 2:**
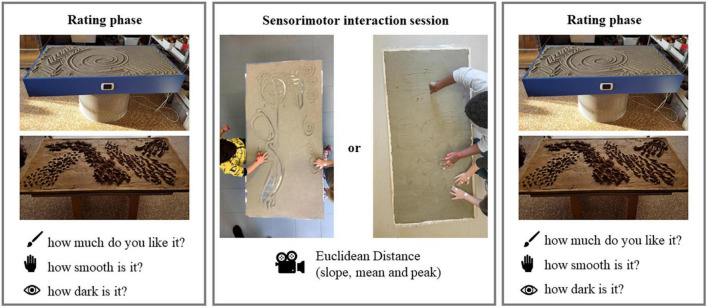
Graphic sketch of the performed experimental protocol. Each rectangle corresponds to a single experimental phase.

The experimental procedure (Figure 2) consisted of two rating phases interspersed with a sensorimotor interaction session. The full experimental session lasted about 15 min. To avert confounding effects, during the 3 months preceding the experimental session, educators did not plan activities involving the use of sand or clay at school. In both rating phases, each child was asked to rate two artifacts laying on two tables and made by two different sculpting natural materials (sand and clay). The ratings were provided using the measurement tool previously described. One artifact, made by sand, showed a series of concentric curves. The second artifact, made by clay, consisted of a series of punctiform depressions. Each artifact was rated according to its tactile (How smooth is it?), visual (How dark is it?), and beauty (How much do you like it?) proprieties. The order of artifacts presentation and questions was balanced between participants. After the child had answered each question, the experimenter measured the score by marking the position where the child had placed the magnetic cursor. Recording participant’s response was performed measuring the distance, in centimeters, between the apex of the triangle and the position of the magnet. The children made the judgments individually and without time limits.

The sensorimotor interaction occurred after the first rating phase and lasted 3 min. It was carried out in a dedicated room by one pair of children at a time, they were asked to freely explore and manipulate the material with their hands. The experimenters gave no other instructions. The children, if they wished, were free to move around the table on which the material was distributed. The tables where artifacts were presented for the rating phases were the same size as the tables where sensorimotor interaction took place. Either sand or clay was placed on the table. Half of the children exclusively interacted with sand, whereas the other half exclusively manipulated clay. A camera was placed on the ceiling above the table to capture children’s hand movements during exploration/manipulation. For each child, colored markers were placed on her/his wrist, index finger, and thumb of both hands. The video recorded during the sensorimotor interactions were then processed with Tracker Video Analysis and Modeling Tool 6^2^ allowing the computation of kinematic and dynamic models of point mass particles in 2D videos.

#### 2.3.3. Statistical analyses

**FIGURE 3:**
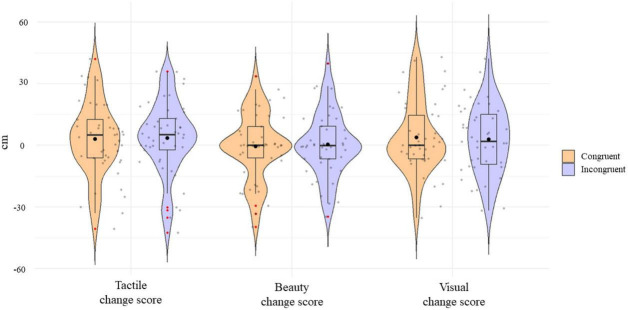
Violin plots showing tactile, beauty and visual change scores obtained in response to the Congruent (orange) and Incongruent (purple) conditions. Black dots indicate the mean values, bold horizontal black lines mark the median values, rectangles identify the interquartile ranges, and the colored areas show scores densities.

The change scores between the ratings given to the material before and after the sensorimotor interaction session were calculated for each question and each material. The change score was calculated as a differential score (i.e., post interaction rating—pre interaction rating) so that higher change scores indicated an increment in children evaluation after sensorimotor interaction. This procedure was followed considering judgment similarity in terms of standard deviations (beauty initial rating: *M* = 32.99, ± 11.81; tactile initial rating: *M* = 24.87, ± 13.78; visual initial rating: *M* = 22.35, ± 13.49) and the adoption of a closed scale for responses. The change scores given to the artifact made with the material manipulated by the participant were named as congruent. Conversely, the change scores given to the artifact made with material with which the child did not interact were considered incongruent. Please, refer to Figure 3 for a graphical representation of the change scores between conditions and across questions.

Out of the 3 min of sensorimotor interaction, a kinematic model of the mass point fixed on the child’s right index finger was computed for the middle minute. Then the Euclidean Distance covered by the mass point is estimated for 10 time bins each lasting 6 s. This procedure allowed the computation of the slope and the mean of the Euclidean Distance covered during the entire middle minute by each participant. The slope and mean of the Euclidean Distance represented the variation along time and the average distance covered by participants’ right hand, respectively. Thus, they worked as proxy measures of the amount of the sensorimotor interaction that each child had with the material.

According to the proposed hypothesis, a modulation was expected only for congruent material change scores. Tactile and visual ratings were used as control for which no modulation due to sensorimotor interaction was expected. If sensorimotor interaction plays a role in children’s aesthetic experience, a modulation of the beauty ratings was expected only for beauty congruent change scores, so that the higher the sensorimotor interaction (higher slope and the mean Euclidean Distance values), the higher the beauty change scores.

To test this hypothesis, three mixed-effect models (one for each Question) were run including Condition (Congruent and Incongruent) and Kinematic parameters (Slope and Mean) as fixed effects. Participants were entered as random effect, and participants’ initial ratings were included as covariate. Whenever the interaction between Condition and Kinematic parameters resulted significant, univariate tests were then run to further explore the significant interaction effects.

All analyses were performed using R software^3^ and lme4, Hmisc, simr, and psych packages. For data visualization we used the ggplot2 package.

### 2.4. Results

The model performed on Tactile change score explained 45% of the variance, taking into account the random effect (*R*^2^m = 0.44; *R*^2^c = 0.45). The model revealed a significant effect of participants’ initial tactile ratings used as covariate [χ^2^_(1)_ = 63.35, *p* 0.001]. Univariate test performed to further investigate this effect showed that the higher the participants’ initial tactile ratings, the lower the Tactile change scores (*F*_(1,88)_ = 62.81, *p* 0.001, β = −0.64, *R*^2^_*adj*_ = 0.41, 95% CI [−1.04, −0.63]; initial tactile ratings: *M* = 24.87 cm, SE = 1.45; Tactile change score: *M* = 3.26 cm, SE = 1.88).

The model performed on Visual change score explained 43% of the variance, taking into account the random effect (*R*^2^m = 0.43; *R*^2^c = 0.43). The model revealed a significant effect of participants’ initial visual ratings used as covariate [χ^2^_(1)_ = 49.08, *p* 0.001]. Univariate test performed to further investigate this effect showed that the higher the participants’ initial visual ratings, the lower the Visual change scores (*F*_(1,84)_ = 48.05, *p* 0.001, β = −0.60, *R*^2^_*adj*_ = 0.36, 95% CI [−0.99, −0.55]; initial visual ratings: *M* = 22.35 cm, SE = 1.45; Visual change score: *M* = 3.35 cm, SE = 1.86).

The model performed on Beauty change score explained 43% of the variance, taking into account the random effect (*R*^2^m = 0.43; *R*^2^c = 0.43). The model revealed a significant effect of participants’ initial beauty ratings used as covariate [χ^2^_(1)_ = 48.69, *p* 0.001], as well as, a significant Condition * Mean interaction [χ^2^_(1)_ = 5.02, *p* 0.02]. Univariate test performed to further investigate the effect of initial beauty ratings showed that the higher the participants’ initial beauty scores, the lower the Beauty change score (*F*_(1,88)_ = 47.26, *p* 0.001, β = −0.59, *R*^2^_*adj*_ = 0.34, 95% CI [−1.03, −0.57]; initial beauty ratings: *M* = 33 cm, SE = 1.24; Beauty change score: *M* = −0.08 cm, SE = 1.68).

**FIGURE 4:**
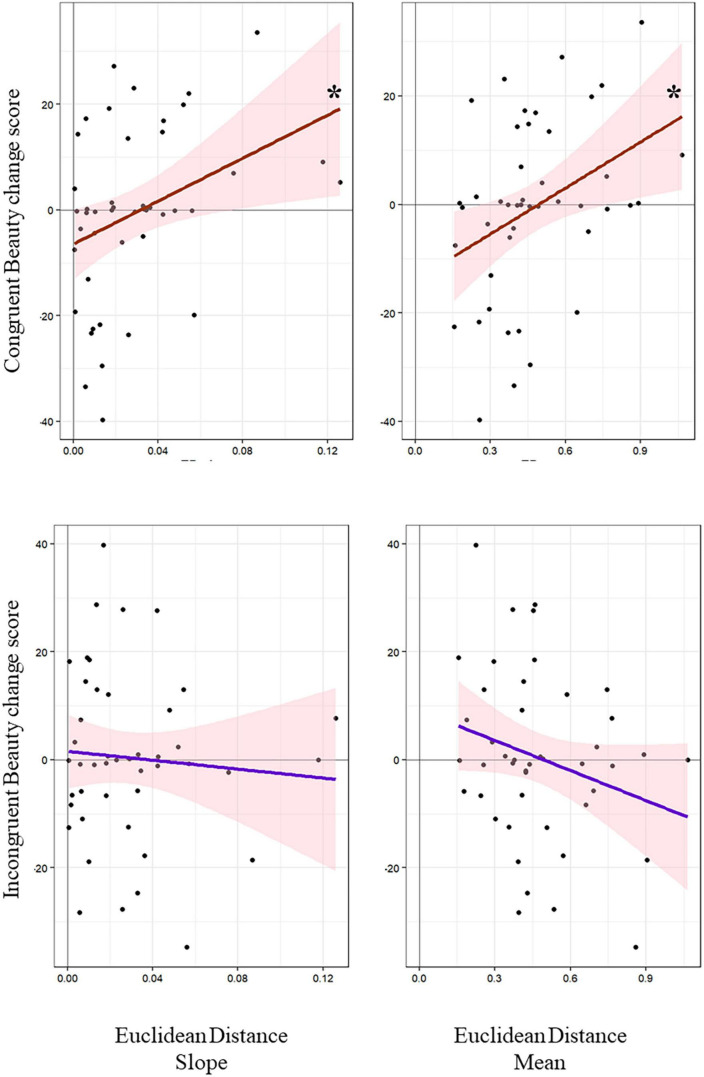
Effect of kinematic parameters (i.e., slope and mean Euclidean Distance values) displayed for Congruent and Incongruent conditions on Beauty change scores *p < 0.05.

Univariate tests (Figure 4) performed to better explore the significant Condition * Mean interaction showed that the higher the mean amount of sensorimotor interaction (i.e., mean Euclidean Distance), the higher the Congruent Beauty change scores (*F*_(1,43)_ = 7.04, *p* 0.01, β = 0.37, *R*^2^_*adj*_ = 0.12, 95% CI [−6.8, 49.8]; Congruent Beauty change scores: *M* = −0.54 cm, SE = 2.44; Mean Euclidean Distance: *M* = 0.47 cm, SE = 0.03). Differently, univariate test performed between the mean amount of sensorimotor interaction (i.e., mean Euclidean Distance) and Incongruent Beauty change scores did not resulted significant (*F*_(1,43)_ = 2.98, *p* 0.09, β = −0.25, *R*^2^_*adj*_ = 0.04, 95% CI [−40.21, 3.11]; Incongruent Beauty change scores: *M* = 0.38 cm, SE = 2.35).

## 3. Discussion

The present study investigated whether sensorimotor experience concurs to the formation of an aesthetic evaluation in preschool children. To accomplish this goal, a group of children rated the tactile, visual and beauty proprieties of two artifacts made by two different sculpting natural materials after having manipulated only one of them. If sensorimotor experience plays a specific role in the formation of an aesthetic judgment, we expected a modulation of the beauty ratings offered to the artifact made by the handled material only.

Looking at the distribution of change scores between conditions and across questions, no substantial modulations can be found (Figure 2). In other words, without considering in the model the amount of sensorimotor experience made by each participant, the manipulation of the material did not modulate any of the explicit judgments made on the artifacts. This null effect is better understood considering the significant and specific modulation that the amount of sensorimotor interaction, operationalized in the kinematic parameters of interest (i.e., slope and mean values of the Euclidean Distance), exerts on the beauty judgment. In fact, the result of the model showed that the greater the sensorimotor interaction, the greater the increment in beauty ratings given by the children on the artifact made by the material previously experienced.

Overall, these results provide us with important insights. The absence of modulation of the explicit ratings apart from the amount of sensorimotor interaction differs from evidence derived from adult populations (Leder et al., 2012; Ticini et al., 2014; Ardizzi et al., 2020a,b). Indeed, in these previous studies, a modulation of aesthetic judgments was visible at the behavioral level without considering the natural inter-individual variation of the included sensorimotor experience. This difference could be due to several factors. On a methodological level, the protocol of the present study involved an active sensorimotor experience separated in time from when the children answered the questions and not a sensory motor simulation offered simultaneously with the beauty judgment. Furthermore, the interaction that the children experienced with the material was free, and as such was extremely variable in terms of the sensorimotor feedback. In contrast, protocols developed on adults required the reproduction of precise gestures (e.g., simulation of ample brush strokes) or facial expressions (e.g., contraction of the corrugator muscle) which was being asked to be performed concurrently with the formulation of the beauty judgment. It is possible that replacing the here proposed free interaction with a controlled gesture reproduction can, even during an early developmental age, trigger the effect at the behavioral level. Another possible explanation could lie in a specific developmental modulation of the link between sensorimotor and aesthetics experience. A previous work has suggested that visual preference for canonical body structures follows non-linear developmental trajectories in preschoolers (Di Dio et al., 2018). Indeed, a recent study showed that motion perception reaches an adult-like level around 8 years of age, whereas form perception continues to develop and reaches an adult-like level around 12 years of age (Benassi et al., 2021). Coherently, Ross and Atkinson (2020) have highlighted that, although the developmental trajectory followed by sensorimotor and body-state simulation is currently unclear, differences between adults and children in specific affective and cognitive processes can be due to a latter’s lack of complete sensorimotor and body-state simulation. Proceeding from the same premises, it is possible to hypothesize that pre-school children have a sensorimotor simulation mechanism that is not yet fully developed and which consequently favors the formation of an aesthetic evaluation to a lesser or more variable extent. In order to confirm or refute this hypothesis, studies integrating the development of aesthetic experience with that of sensorimotor simulation processes in a longitudinal perspective would be necessary.

The significant and specific modulation of beauty judgments associated with the mean amount of sensorimotor interaction, instead, suggests that even in pre-school populations the aesthetic experience is not completely decoupled from its sensorimotor component, supporting, from a developmental perspective, the definition of the aesthetic triad proposed by Chatterjee and Vartanian (2014). It is important to point out that, among the kinematic variables considered, it is the average of movement (mean Euclidean Distance) and not its variation over time (slope of Euclidean Distance) that was significant. This suggests a more general effect of the amount of sensorimotor interaction rather than its variability. Further analyses, with respect to the quality of movements performed, could help to better describe this phenomenon in a child population. Our main result brings previous findings into a broader interpretative framework, emphasizing that also in the case of aesthetic experience, sensorimotor constituents contribute to the development of such high-level cognitive function. The sensorimotor contribution to human cognitive development is not in controversy to date. Numerous studies, for example, have linked sensorimotor experiences to the development of linguistic (Mazzuca et al., 2021) or arithmetic (Barrocas et al., 2020) skills in children. This is, however, the first time that this relationship has also been clearly highlighted in preschoolers for the formation of aesthetic judgment. Our results can also be interpreted in line with the theories on the role of sensorimotor development in children elaborated by Vygotsky (1978); Newman and Holzman (2013), and Klimkowski (2020) who believed that the acquisition of motor skills was closely related to the development of higher mental processes. He argued that children’s early motor behaviors, such as grasping and reaching are essential precursors to later cognitive development and that aesthetic appreciation is an important aspect of children’s development and plays a significant role in their emotional and cognitive growth. He supposed that children’s early experiences with art, music, and literature help to stimulate their imagination, creativity, and critical thinking skills. His broader theoretical framework for the development of children’s cognitive, emotional, and social skills also addresses the interplay between aesthetics and sensory motor skills. According to Vygotsky (1978) sociocultural theory, children’s development is shaped by their social and cultural environment, children learn through interaction with others and the tools and practices of their culture. In this context, aesthetic appreciation and motor skills are interrelated and mutually supportive. Important pedagogical remarks can thus be further opened up. As already pointed out (Swann, 2008), preschoolers’ development progresses from children’s exploratory actions on the objects and materials to their increasingly more complex explorative relationships to support a range of emerging representations props of symbolic play, letters of the alphabet, and also, aesthetic experience. These actions provided foundations of learning and prefigure later phases in bodily and cognitive development. Therefore, aesthetic curriculum for young children should tap into children’s sensorimotor experiences by encouraging them to structure knowledge-building activities in ways that are the natural extensions of the sensorimotor experiential knowledge they already possess. It is important to highlight that aesthetics is often considered as limited to the study of art, but in contemporary educational theory and practice it has come to mean a variety of rather different things, such as sensory education, beauty appreciation, social education, affective and moral development (Carr, 2013).

This study has some limitations to be considered. First, we explored the role of sensorimotor experience in a limited population of 5 years old children. Longitudinal studies are needed to better understand the developmental trajectory of sensorimotor contribution to aesthetic experience. Furthermore, we had restricted the evaluation of aesthetic experience to a beauty judgment. Although this is a frequently used proxy to study aesthetic experience, it is plain that aesthetic experience, even at pre-school age, extends far beyond the mere judgment of liking to encompass emotional and reward dimensions. In fact, most likely the manual interaction with the material was a multidimensional pleasant experience for the children that was reflected in the increased score they gave to the beauty judgment. Coherently, we cannot rule out an addictive effect of the hedonic feelings elicited by the sensorimotor experience on the modulation of Congruent Beauty ratings. Lastly, the present protocol directly tests the role of an active free sensorimotor experience rather than a true sensorimotor simulation. However, proceeding from the present results, it will be possible to design protocols to evaluate also in children the contribution of sensorimotor simulation on aesthetic judgment similarly to what has been more commonly tested in adults.

In conclusion, the overarching suggestion of the present study is that one (though not the only) important avenue for children education lies in the vital relevance of sensorimotor experiences to the cultivation of a wealth of virtuous resources and skills that can be invested by children outside and inside educational contexts during development.

## Data availability statement

The raw data supporting the conclusions of this article will be made available by the authors, without undue reservation.

## Ethics statement

The studies involving human participants were reviewed and approved by the Institutional Review Board of the University of Parma (Prot. 0009293). Written informed consent to participate in this study was provided by the participants’ legal guardian/next of kin. Written informed consent was obtained from the individual(s) for the publication of any identifiable images or data included in this article.

## Author contributions

MA and MU conceptualized the study together with CG and EM. MA, AM, FF, and MU collected the data. MA, AM, FF, and SU analyzed the behavioral and kinematic data and performed the statistical analyses. SU gave valuable expert support for interpreting the kinematic results. CG and EM have made important contributions to interpreting the impact of results in education and pedagogy. MA and MU conceptualized the manuscript. MA and AM wrote the manuscript with contributions from CG, EM, FF, MU, and SU. All authors approved the final version of the manuscript and read and agreed to the published version of the manuscript.

## Footnotes

1
https://v3.glimmpse.samplesizeshop.org/#

2
https://physlets.org/tracker/

3
https://www.r-project.org/
